# Virginia’s Response to the Nursing Home COVID Action Network

**DOI:** 10.1093/geroni/igab046.1899

**Published:** 2021-12-17

**Authors:** Leland Waters, Anne Rhodes, Shannon Arnette, Dan Bluestein, Emily Ihara, Megumi Inoue, Catherine Tompkins

**Affiliations:** 1 Virginia Commonwealth University, Richmond, Virginia, United States; 2 George Mason University, Fairfax, Virginia, United States

## Abstract

The Virginia Geriatric Education Center’s GWEP recruited 195 of Virginia's 273 eligible nursing homes, using two Project ECHO Nursing Home Training Centers located at George Mason University and Virginia Commonwealth University. These sessions promoted collaboration, allowed for sharing of successes and challenges, and nurtured quality improvement projects. Our next steps are to survey Virginia’s nursing homes to see if they are interested in future ECHO sessions with other topics. We plan to share these results with the Institute for Healthcare Improvement so that we may be able to continue to enhance this national network of Training Centers with faculty and staffing dedicated to quality assurance and performance improvement. The program has initiated new collaborations with nursing homes across many healthcare disciplines, strengthened connections between nursing homes and research institutions, and will help foster innovative ways to collaborate in this post-pandemic virtually connected world.

